# Enhancing Neurogenesis of Neural Stem Cells Using Homogeneous Nanohole Pattern-Modified Conductive Platform

**DOI:** 10.3390/ijms21010191

**Published:** 2019-12-26

**Authors:** Yeon-Woo Cho, Da-Seul Kim, Intan Rosalina Suhito, Dong Keun Han, Taek Lee, Tae-Hyung Kim

**Affiliations:** 1 School of Integrative Engineering, Chung-Ang University, Seoul 06974, Korea; healerforyou@naver.com (Y.-W.C.); kim_daseul@hanmail.net (D.-S.K.); intanrosalinasuhito@gmail.com (I.R.S.); 2 Department of Biomedical Science, College of Life Sciences, CHA University, 335 Pangyo-ro, Bundang-gu, Seongnam, Gyeonggi 13488, Korea; dkhan@cha.ac.kr; 3 Department of Chemical Engineering, Kwangwoon University, Wolgye-dong, Nowon-gu, Seoul 01899, Korea; tlee@kw.ac.kr; 4 Integrative Research Center for Two-dimensional Functional Materials, Institute of Interdisciplinary Convergence Research, Chung Ang University, Seoul 06974, Korea

**Keywords:** nanotopography, laser interference lithography, neural stem cell, neuronal differentiation

## Abstract

Biocompatible platforms, wherein cells attach and grow, are important for controlling cytoskeletal dynamics and steering stem cell functions, including differentiation. Among various components, membrane integrins play a key role in focal adhesion of cells (18–20 nm in size) and are, thus, highly sensitive to the nanotopographical features of underlying substrates. Hence, it is necessary to develop a platform/technique that can provide high flexibility in controlling nanostructure sizes. We report a platform modified with homogeneous nanohole patterns, effective in guiding neurogenesis of mouse neural stem cells (mNSCs). Sizes of nanoholes were easily generated and varied using laser interference lithography (LIL), by changing the incident angles of light interference on substrates. Among three different nanohole patterns fabricated on conductive transparent electrodes, 500 nm-sized nanoholes showed the best performance for cell adhesion and spreading, based on F-actin and lamellipodia/filopodia expression. Enhanced biocompatibility and cell adhesion of these nanohole patterns ultimately resulted in the enhanced neurogenesis of mNSCs, based on the mRNAs expression level of the mNSCs marker and several neuronal markers. Therefore, platforms modified with homogeneous nanohole patterns fabricated by LIL are promising for the precise tuning of nanostructures in tissue culture platforms and useful for controlling various differentiation lineages of stem cells.

## 1. Introduction

Neural stem cells (NSCs) are multipotent cells, which can differentiate into neuronal cells or glial cells and exist in diverse areas of the central neural system (CNS) [1,2]. In previous studies, mammalian neurodevelopment or neurogenesis was proven to occur not only during embryonic development, but also in the adult nervous system [2,3]. Thus, NSCs were used to elucidate mechanisms of neural development that were relatively less understood. Above all, owing to their differentiation potential, NSCs have been recognized as a promising candidate for stem cell therapies in neurodegenerative diseases, including Alzheimer′s disease, Parkinson′s disease, and stroke [4,5,6]. Considering the efficacy of stem cell therapies, it is crucial to increase the differentiation efficiency of stem cells, which refers to its differentiation into preferred cell types [7,8].

In fact, various cellular functions of native cells in real tissues are regulated through interactions within their own microenvironment, a dynamic environment consisting of extracellular matrices (ECMs), where specific cells are provided unique physical cues or information (e.g., roughness, texture, and stiffness) [9,10,11,12]. The interactions between cells and their ECMs providing physical cues act as mechanical stimuli, by which various cell signaling pathways and their downstream pathways are up or downregulated, referred to as mechanotransduction [13]. In addition, mechanotransduction begins from focal adhesions, which function as a physical link to the ECM and influences various cell downstream signaling pathways involved in stem cell fate, such as differentiation [14,15,16,17,18]. Specifically, it has been reported that NSCs can sense tension in its cellular membrane through mechanotransduction. Many studies on controlling the differentiation of neural stem cells in modified culture systems that provide specific physical cues have been reported [19,20].

Various nanobiomaterials (e.g., nanoparticles, porous polymers, and nanopatterning) have proven to be favorable in increasing cell spreading and focal adhesion through supportive topographical cues [21,22,23,24,25,26,27]. In this regard, the enhancement of the specific differentiation of various types of stem cells could also be influenced by the advantageous properties of biocompatible nanomaterials [28,29,30,31,32,33]. However, the nanoparticle-based approach used to regulate stem cell fate has some limitations in therapeutic application due its potential for bioaccumulation, a phenomenon in which gradual accumulation of substances occurs in the body, causing toxicity to stem cells [34,35,36]. Additionally, it is difficult to precisely fabricate porous polymers with a homogeneous nanostructure. In contrast, homogenous nanostructures can be fabricated with several nanopatterning techniques, as well as techniques that can precisely control the dimension of nanostructures, including the size, shape, and pitch of nanopatterns [37,38,39,40,41].

To date, several techniques (e.g., photolithography, nanoshaving, nanografting, dip-pen nanolithography) have been revealed, and are widely used to fabricate nanopatterns [42,43,44,45,46,47,48,49]. Among these, photolithography, a type of lithography that generates patterns with known sequential processes (e.g., cleaning, photoresist (PR) coating, exposure, development, and etching), has been dominant. This is due to its clear advantages for nanopatterned fabrication, including the quality of nanopatterns generated, high variability in size/shape of nanostructures, and ease of replication [50]. However, such lithographic techniques also have clear limitations: (i) difficulties in manufacturing photomasks, (ii) high cost for photomask production, and (iii) difficulties in operation. Such disadvantages are especially critical for stem cell applications as cell membrane receptors; integrins responsible for cell adhesion, specifically, are 20–26 nm in size and, thus, highly sensitive to small changes in the size/gap/shape of nanopatterns modified on the substrate [51,52]. Hence, a technique/platform that provides high flexibility in the size of nanopatterns, with well-defined and organized arrangements, is essential to control stem cell differentiation via physical (i.e., insoluble) cues.

**Figure 1:**
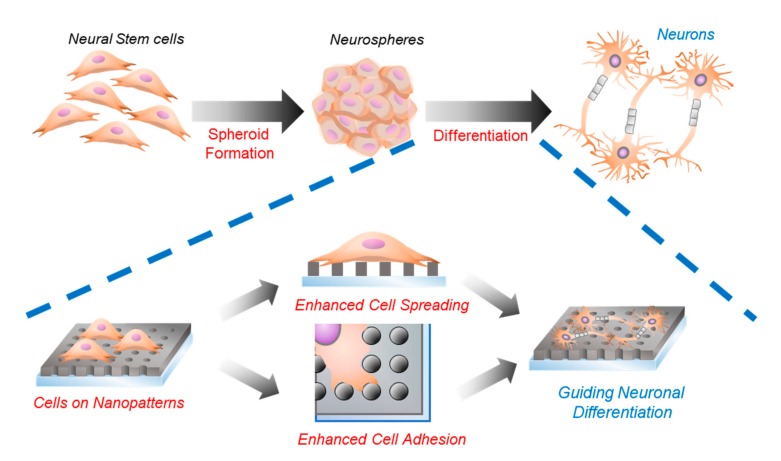
A schematic illustration of the topographical effects of nanopatterns on neuronal differentiation of mouse neural stem cells (mNSCs). Blue line indicates the role of nanostructures on neuronal differentiation.

To this end, we report a platform modified with homogeneous polymeric nanohole patterns that enables the enhancement of the neuronal differentiation of mouse neural stem cells (mNSCs). Considering the size of the cells (10–100 µm), for the first time, we used laser interference lithography (LIL), a technique proven effective in large-scale fabrication of periodic nanopatterns, for controlling the differentiation of mNSCs. Homogeneous nanohole pattern arrays (HNPAs) of three different sizes (500 nm, 700 nm, and 900 nm in hole size) were generated on conventional glass substrates, and mNSCs were further cultured and differentiated into neuronal cells. Among the varying nanopatterned geometry of the platforms, we observed that the 500 nm nanohole platform showed the best performance in terms of several stem cell functions (e.g., adhesion, spheroid formation, and cell growth), which ultimately resulted in enhanced neuronal differentiation of mNSCs, based on the mRNA and protein marker expressions (e.g., TuJ-1, NeuroD1, and MAP2). This study revealed that the geometry of nanopatterns can be easily varied using LIL, and that it is a highly promising approach for optimizing the structure/morphology of homogeneous nanopatterns to effectively enhance cell functions (e.g., cell spreading and adhesion) and guide neuronal differentiation of stem cells (Figure 1).

## 2. Results and Discussion

### 2.1. Fabrication and Characterization of Nanohole Pattern

**Figure 2:**
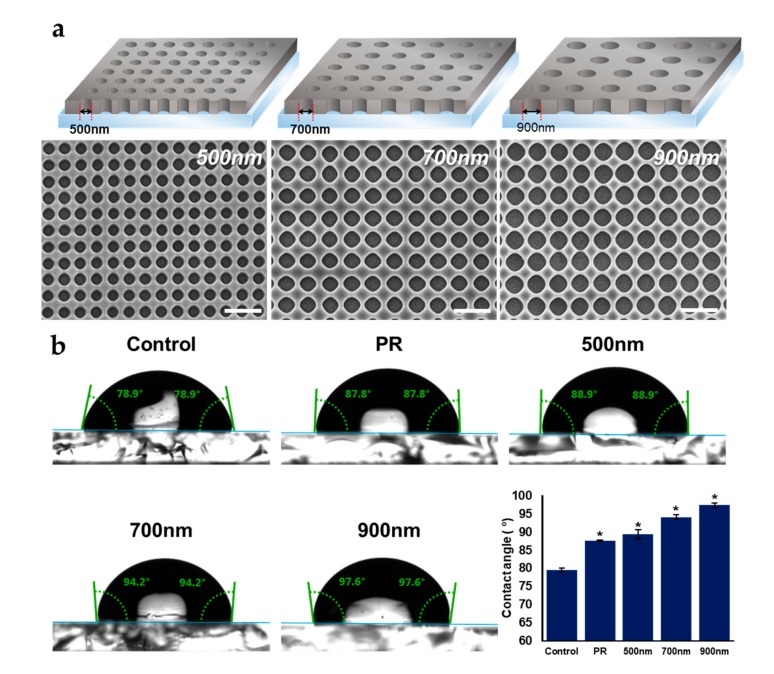
Surface characterization of substrates. (**a**) Field emission scanning electron microscopy (FE-SEM) images of homogeneous nanohole pattern arrays (HNPAs) with hole size of 500 nm, 700 nm, and 900 nm, (**b**) Contact angle measurements for all groups. Control = bare indium tin oxide (ITO) substrate; * Compared to control group, *p* < 0.05, *n* = 3; unpaired student′s *t*-test, scale bar = 2 µm.

As mentioned above, homogeneous HNPAs can influence various cell functions, including cell spreading, adhesion, and neural differentiation, by providing topographical and physical cues; it plays a crucial role as its own ECM. In fact, different conditions of nanopatterns (e.g., pattern shape, pattern size, and pitch size) generate various surface topographies, and distinctively affects cell fate regulation [22,41]. In order to investigate the effects of pattern size on cellular processes and achieve optimal condition of HNPAs, we designed three HNPAs of different hole sizes (500 nm, 700 nm, and 900 nm), where the gap between each pattern was determined by a fixed value (300 nm). Additionally, it has been proven that homogenous nanostructures enhance cell adhesion and growth through integrin clustering [53]. Moreover, electrical stimulation is known to influence the proliferation and differentiation of NSCs. Conductive nanomaterials could be useful for applying electrical pulses to cells, to further increase the differentiation of NSCs [54,55,56]. For this reason, we tried to fabricate HNPAs on conductive indium tin oxide (ITO)-coated glass, using LIL processes. Figure 2a shows successful fabrication of HNPAs with the diameters of 500 nm, 700 nm, and 900 nm, and nanohole size variations of less than 20%. The gap and height in the pattern area (1.2 × 1.2 cm) were subsequently confirmed as 300 nm and 750–800 nm, respectively. Pattern height was precisely controlled by plasma etching, and the total number of nanoholes in the entire pattern area of the HNPAs with hole sizes of 500 nm, 700 nm, and 900 nm, were 2.25 × 10^8^, 1.44 × 10^8^, and 1.00 × 10^8^, respectively.

Based on previous studies that concluded the range of sites for cell adhesion is lower than 200 nm, HNPAs with hole size of 500 nm (HNPA-500 nm) as the smallest nanoscale-pattern might be optimum for cell adhesion and spreading [57]. Total contact area, referred to as an entire non-patterned area in HNPA, where cells directly make contact with the surface of HNPAs with hole sizes of 500 nm, 700 nm, and 900 nm, were 1.00 cm^2^, 0.89 cm^2^, and 0.80 cm^2^, respectively. Our hypothesis is primarily based on the larger contact area generated on the HNPA-500 nm substrate, compared to other HNPAs groups. Since it was reported that improved cell adhesion can enhance differentiation and proliferation, neural differentiation should increase in the HNPA-500 nm substrate [58]. The total perimeter of nanoholes on the entire patterned area of HNPA-500 nm, HNPA-700 nm, and HNPA-900 nm, were calculated to be 3.35 × 10^2^, 3.17 × 10^2^, and 2.83 × 10^2^, respectively. Given the enhancement of neuronal differentiation, increased surface area of HNPAs would have a greater effect on neuronal differentiation. In addition to the confirmation of nanohole generation, the hydrophobicity, which is a critical factor for cell adhesion and growth, was investigated for all fabricated substrates. As shown in Figure 2b, PR modifications, both for PR-modified substrate and HNPAs, increased hydrophobicity when compared with bare ITO substrate (control). Interestingly, owing to the difference in the diameter of nanohole size, the contact angle of HNPA-500 nm was found to be 4.92% and 9.29% lower than in HNPA-700 nm and HNPA-900 nm, respectively. This indicates that HNPA-500 nm is less hydrophobic than other substrates, and can thus be better for enhancing cell adhesion on the nanohole surfaces over other HNPAs with different nanohole sizes. Hence, we hypothesized that the HNPA-500 nm, among all substrates including control (normal tissue culture plate), non-patterned group (Photoresist-coated substrates, PR), and HNPAs, would be excellent for the improvement of cellular functions and ultimately cell fate determination.

### 2.2. The Effects of Nanohole Patterns on mNSCs Adhesion and Growth

**Figure 3:**
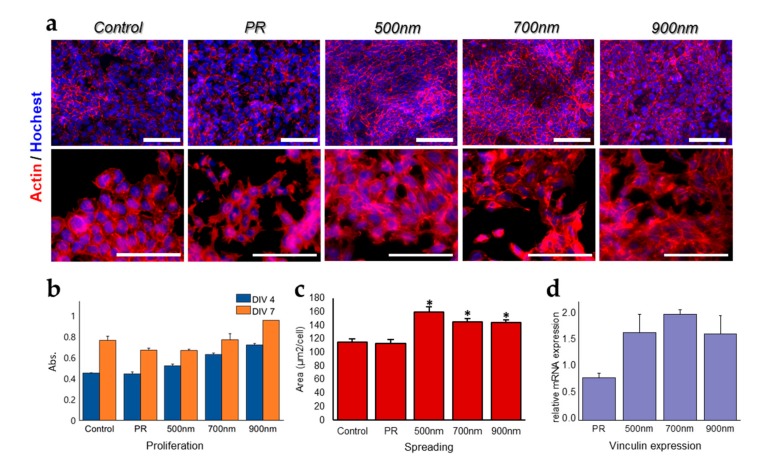
Confirmation of cell adhesion and spreading of mNSCs cultured on all substrates. (**a**) Immunofluorescence staining images of mNSCs with phalloidin, Hoechst, and vinculin after 7 d of proliferation on HNPAs and non-patterned groups (top panel). Enlarged immunofluorescence staining images of mNSCs stain with phalloidin and Hoechst after 7 days of proliferation on HNPAs and non-patterned groups (bottom panel). (**b**) Number of cells cultured on HNPAs and non-patterned groups after 4 d and 7 d of proliferation. (**c**) Cell spreading, calculated based on three different F-actin/Hoechst staining images for each HNPA and non-patterned group. (**d**) RT-qPCR results for vinculin indicating focal adhesion of mNSCs. Control = bare ITO; the results were normalized to control group; * Compared to control group, *p* < 0.05, *n* = 3, unpaired student’s *t*-test; scale bar = 100 μm.

Focal adhesion is one of the major cellular regulators of stem cell growth and differentiation. This is due to its role in the interaction of the actin cytoskeleton with its extracellular matrix (ECM) by binding the integrin receptor and actin filaments via actin-binding proteins (e.g., alpha actinin, vinculin, and talin), and direct involvement in the reorganization of cytoskeletal filaments [59,60,61,62]. As previously mentioned, HNPAs are capable of improving cell adhesion and growth through the reorganization of the cytoskeleton. Therefore, in this study, we affirm that HNPAs are powerful for accelerating stem cell growth and differentiation [53,63,64]. According to this premise, we determined the correlation of cell adhesion, spreading, and growth with neural differentiation of mNSCs. Figure 3 shows cell spreading, adhesion, and growth of mNSCs cultured on HNPAs and non-patterned groups. To prove the effect of HNPAs on mNSC adhesion, cells grown on all groups were stained with phalloidin (exhibits F-actin arrangement) and Hoechst (binds to the nucleus) (Figure 3a). Upon actin staining, cell spreading area of all HNPAs were increased when compared to that of non-patterned substrates, including the control and PR groups. In the case of quantification results for cell spreading, HNPA-500 nm, HNPA-700 nm, and HNPA-900 nm were 40.1%, 38.6%, and 33.6% higher than that of control, respectively (Figure 3c). These results indicate that HNPAs triggered cell spreading through cytoskeleton expansion formed by actin microfilaments, compared to non-patterned groups. Subsequently, mRNA expression level of vinculin, as an adhesion marker on all groups, was carried out using RT-qPCR to assess the effect of HNPAs on focal adhesion (Figure 3d) [65,66]. Based on the data, vinculin expression was higher on all HNPAs groups than on non-patterned groups, indicating that HNPAs enhance focal adhesion via increased formation of actin-binding proteins. Furthermore, the cell viability rate was analyzed using the cell counting kit-8 (CCK-8) assay at 4 d and 7 d of growth (Figure 3b). We found that cell growth for 4 d was enhanced on all HNPAs, when compared with the control and PR groups; whereas cell growth for 7 d was enhanced on HNPA-900 nm only among all HNPAs, compared to non-patterned groups. Altogether, it can be concluded that HNPAs are promising for the improvement of crucial cellular functions, such as cell adhesion, spreading, and proliferation, which is consistent with the results of previous studies [53,67].

**Figure 4:**
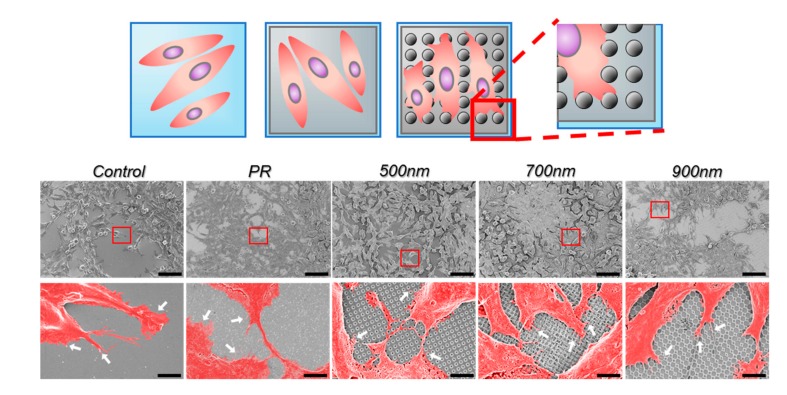
Pseudo-colored scanning electron microscopy (SEM) images of mNSCs showing cell spreading and filopodia after 4 d of cultivation on HNPAs with different hole size and non-patterned groups. Arrows indicate the filopodia projections toward nanoholes on the underlying substrates. Scale bars in top panel = 50 µm; Scale bars in bottom panel = 5 µm; Control = bare ITO substrate; red boxes: enlarged area; white arrows: filopodia.

In addition, to investigate the interaction between the HNPAs and mNSCs, SEM images were further obtained (Figure 4). We focused on the filopodia formations that extended from lamellipodium, since filopodia are known to be critical in several cell functions, especially cell focal adhesion, migration, and even growth [68]. Based on the pseudo-colored high magnification SEM images, we found that mNSCs in all groups were well spread on the surface. Interestingly, filopodia projections toward substrates were found to be highly enhanced around nanohole patterns regardless of the sizes of nanohole patterns, as shown in Figure 4. This is completely different from the typical flat surface (e.g., control and PR group) showing randomly distributed filopodia projections from lamellipodium. Taken together, it can be concluded that HNPAs are promising for the improvement of crucial cellular functions, such as cell adhesion, spreading, and proliferation, which is consistent with the results of previous studies [53,67].

### 2.3. Neurosphere Formation of mNSCs on Nanohole Pattern Arrays

**Figure 5:**
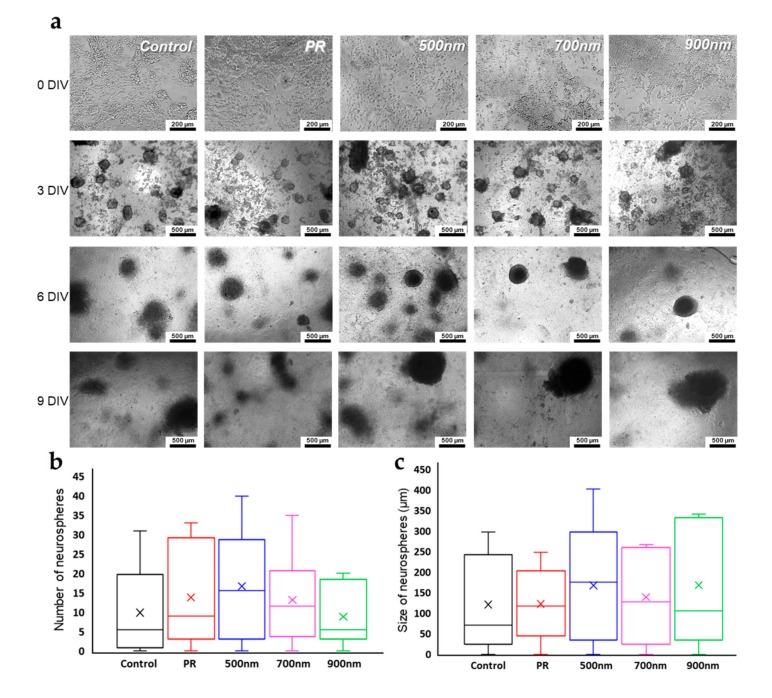
Neurosphere formation during cultivation of mNSCs. (**a**) The optical microscopic images of mNSCs cultured on HNPAs and non-patterned groups. (**b**) Number of the neurosphere of nNSCs cultured on HNPAs and non-patterned groups. (**c**) Size of the neurosphere of nNSCs cultured on HNPAs and non-patterned groups. Scale bar in top panel = 200 µm; Scale bar in other panels = 500 µm; *n* = 3.

Neurospheres are three-dimensional clusters of neural stem cells, whereas neurites are projections from neural stem cells, the outgrowth of which occurs during neural development [69]. Both neurosphere formation and neurite outgrowth have been recognized as critical events in neurogenesis. Specifically, neurosphere formation during the differentiation of neural stem cells into mature neuronal cells or glial cells has been reported to produce its own specific proteins, which are further involved in neuronal differentiation. Additionally, neurospheres contain various types of neural lineage cells, including neural progenitor cells or immature neural cells; differentiated mature cells are generated from neurospheres [69,70]. In the mammalian central nervous system, neurons are polarized, and a single axon protrudes from the cell body to several dendrites that facilitate cell–cell interactions. Neuronal polarization is initiated via axon specification; neurite outgrowth, known to differentiate into axons or dendrites, is necessary for axon specification [71]. In this section, we specifically focus on neurosphere formation of mNSCs cultured on all groups of HNPAs substrates. To evaluate the effects of HNPAs on both parameters of neuronal differentiation, we cultured and induced neural differentiation of mNSCs for 14 d with its differentiation factor, retinoic acid (RA) (Figure 5a). After 3 days of differentiation, the neurosphere formation, which is a typical process for neuronal differentiation of mNSCs, was clearly observed on both non-patterned substrates and HNPAs. However, the number and size of neurospheres on each substrate were different, as shown in Figure 5b,c. Specifically, the median size of neurospheres on HNPAs-500 nm and HNPAs-700 nm were 69.5% and 33.9% higher than control, respectively. Additionally, the maximum size of neurospheres on HNPA-500 nm and HNPA-700 nm increased by 29.0% and 12.9%, respectively, as compared to control, indicating that the maximum size of neurospheres could be achieved on the HNPA-500 platform. This suggests that the formation of neurospheres was enhanced on the HNPA-500 nm substrate. Notably, the average number and size of neurospheres on the PR group, were higher and smaller than those of the HNPA-900 nm group, respectively. Considering the aggregation upon adjacent neurospheres through cell–cell interaction, we can conclude that HNPAs may also facilitate the interaction between each neurosphere, through enhanced cell adhesion and spreading.

### 2.4. Investigations of the Effects of Nanohole Pattern Arrays on Neuronal Differentiation of mNSCs

**Figure 6:**
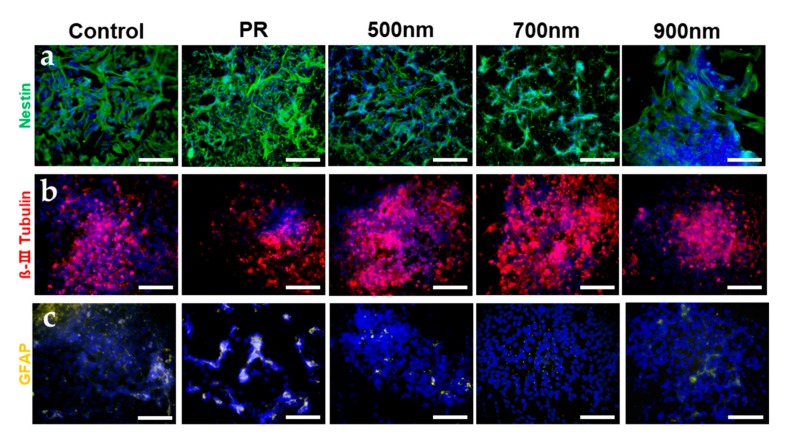
Immunofluorescence staining upon neural differentiation of mNSCs with (**a**) nestin as an undifferentiation marker, and (**b**) β-III Tubulin (beta -III; Tubulin) and (**c**) GFAP (Glial fibrillary acidic protein) as differentiation markers. Pseudo-colored fluorescent images of mNSCs were done for Figure C. Control = bare ITO substrate; scale bar = 100 μm.

In addition to the evidence showing that HNPAs have an important role in increasing cell adhesion, spreading, and neurosphere formation, we also investigated the effects of HNPAs on the neural differentiation of mNSCs. First, cells were cultured in growth medium and subsequently treated with differentiation medium containing RA, known as a neural differentiation factor of mNSCs. After confirmation of morphological changes, like the formation of neurites from neurospheres which indicates neuronal maturation, mNSCs cultured on all substrates were stained to visualize the expression of nestin as a neural stem cell marker, β-III tubulin as an immature neuron marker, and glial fibrillary acidic protein (GFAP) as an astrocyte marker, in order to confirm the direction of neural differentiation (Supplementary Figure S1, Figure 6). According to immunostaining data, the average level of nestin was decreased 1.56 times in the HNPAs, compared to in the control and PR groups. This demonstrated that neural differentiation of mNSCs cultured on HNPAs may increase, since nestin is a parameter that indicates an undifferentiation state in neural stem cells (Figure 6a). On the other hand, the level of β-III Tubulin showed no apparent differences in mNSCs cultured on all patterned and non-patterned groups, and GFAP was barely expressed in all HNPAs; unlike the control, where all these phenomena led to the assumption that neural differentiation into astrocytes was difficult to facilitate in HNPAs (Figure 6b,c).

**Figure 7:**
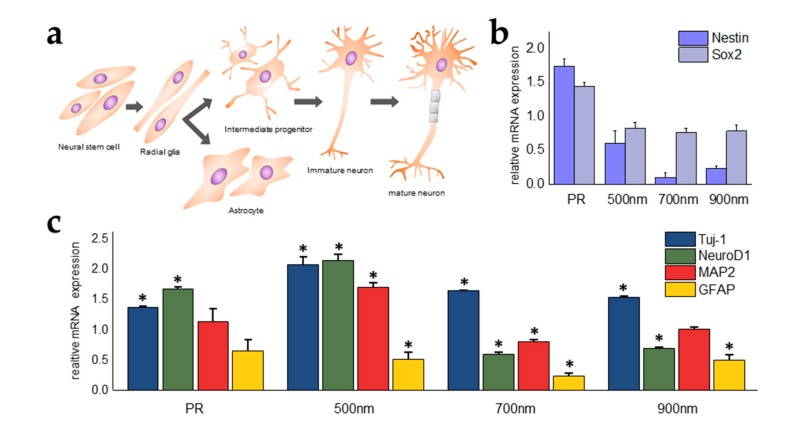
qPCR analysis of neural differentiation of mNSCs (**a**) A schematic diagram representating neural differentiation processes from neural stem cells into differentiated cells (mature neurons or astrocytes). RT-qPCR data for (**b**) undifferentiation markers (Nestin and SOX2), (**c**) neuronal markers (TuJ-1, NeuroD1, and MAP2) and glial markers (GFAP). Control = bare ITO substrate; the results were normalized to control group; * *p* < 0.05, *n* = 3, unpaired student’s *t*-test.

In fact, neural stem cells are known to differentiate into neuronal cells or glial cells, and achieve maturation via intermediate progenitor and immature cells (Figure 7a). While differentiation and maturation occur in neural stem cells, various cellular signaling pathways are involved in these processes. Therefore, to confirm the effects of nanohole size on neural differentiation, mRNA expression levels of two markers for undifferentiated cells (e.g., nestin, sex determining region Y-box 2 (SOX2)) and several markers for differentiated neural lineage cells (e.g., class III beta-tubulin (TuJ1), GFAP, Neuronal Differentiation 1 (NeuroD1), and Microtubule associated protein 2 (MAP2) were analyzed using RT-qPCR (Figure 7b,c). The expression level of nestin in cells grown on HNPAs was much lower than that in non-patterned groups (Figure 7b); especially in HNPA-700 nm, where the decrease was greater than five times that of the control group. Similarly, the expression level of SOX2, a marker downregulated during neural differentiation, was also lower in HNPAs than in non-patterned groups, showing that the level of undifferentiation decreased and that neural differentiation may increase in HNPAs. To confirm whether the patterned substrate enhanced neural differentiation or directed specific differentiation (e.g., neuronal differentiation, into neurons; glial differentiation, into astrocytes), the expression levels of neural differentiation in HNPAs were compared with those in non-patterned groups (Figure 7c). The expression levels of TuJ1, an immature neuron marker, were 2.06, 1.63, 1.52, and 1.36-fold higher on HNPA-500 nm, HNPA-700 nm, HNPA-900 nm, and PR substrates, respectively, than that of control. Remarkably, unlike Tuj1, MAP2, a representative marker of matured neurons, was found to be highly enhanced on HNPA-500 nm (1.69-fold higher than control); whereas the same marker on both the HNPA-700 nm and HNPA-900 nm were 0.79 and 0.99-fold lower than control, respectively. In addition, the trend of NeuroD1 expression between each group was similar to that of MAP2, which is consistent with previous studies reporting the important role of NeuroD1 in neuronal maturation. The expression levels of GFAP in all groups were found to be inhibited, proving that mNSCs cultured on HNPAs were not successfully differentiated into astrocytes. Taken together, selective neuronal differentiation as well as greatly increased maturation of neurons, were confirmed in the HNPA-500 nm. This strongly supports our hypothesis mentioned in Section 3.1, that the HNPA-500 nm is highly suitable for the enhancement of cell functions, including differentiation.

## 3. Materials and Methods

### 3.1. Materials

Indium tin oxide glass was purchased from U.I.D (Cheongju, Korea). All chemical materials used for laser interference photolithography-based nanopatterning, including photoresist (AZ nLof-2020), developer (AZ 300MIF developer), and thinner (AZ 1500 thinner), were obtained from MicroChemicals GmbH (Germany); and hexamethyldisilazane (HMDS) was purchased from Sigma-Aldrich (Germany). Gibco Minimum Essential Medium (MEM) Alpha and antibiotics were purchased from Invitrogen (USA). Several materials necessary for cultivation and differentiation induction, poly-L-lysine (PLL), L-glutamine, RA, and 10% neutral buffered formalin, were obtained from Sigma–Aldrich (Germany). Dulbecco’s phosphate buffered saline (DPBS) was purchased from Gibco (Massachusetts, USA). Monoclonal anti-mouse/rat nestin antibody, anti β-III tubulin antibody, NorthernLights-conjugated anti-mouse IgG emitting 493 nm and 557 nm were purchased from R&D Systems (Minneapolis, MN, USA). Alexa Fluor-conjugated anti-mouse IgG emitting 568 nm was purchased from Abcam (Cambridge, UK). A CCK-8 was obtained from Dojindo Laboratories (Kumamoto, Japan) to test cell viability. All chemicals used in this research were of reagent grade.

### 3.2. Fabrication of HNPAs and Surface Characterization

The HNPAs were fabricated using laser interference photolithography with a Lloyd’s mirror interferometer. First, ITO glass (10 Ω/cm^2^, 0.5 mm thickness) was cleaned with 1% Triton X-100 solution, deionized water, and 70 % ethanol for 20 min. Sterilized ITO glass was coated with HMDS and PR diluted with thinner (6:4), using the spin coating method. This HMDS/PR-coated substrate was then baked on a hot plate (soft bake). The soft-baked substrate was then exposed twice to ultraviolet (UV) light (wavelength = 325 nm, power output = 10 mW) through the Lloyd’s mirror interferometer. After the first exposure, the substrate was rotated 90° clockwise and exposed again to the UV light to fabricate the hole patterns. The UV-exposed substrate was then baked again (post-exposure bake), followed by a developing step with developer. The desired diameter of the nanohole and pitch of pattern were calculated by adjusting the incident angle of the laser light to the mirror; the theoretical calculation was as follows:

(1) $$\Lambda =\lambda_{laser}/2\sin\theta$$

where, Λ, λ_laser_, and θ are the pitch of pattern (nm), wavelength of the UV laser (325 nm), and the incidence angle (°), respectively. All substrates were characterized using field emission scanning electron microscopy (FE-SEM, Carl Zeiss, Germany). In order to assess hydrophobicity of all substrates used in this study, contact angles of the substrates were analyzed using a drop shape analyzer (DSA25, A. Krüss Optronic GmbH, Germany).

### 3.3. Cell Culture and Analysis of Cell Growth, Spreading, and Adhesion of mNSCs

First, a chamber was attached onto the pattern substrate using polydimethylsiloxane (PDMS), a biocompatible glue that does not have any adverse effects on cell growth, to culture mouse neuroectodermal stem cells (NE-4C). The NE-4C passage 6 was obtained from Sogang University (Korea) and cultured in MEM alpha, supplemented with 4 mM L-glutamine, 10% fetal bovine serum (FBS), and 10% antibiotics. All substrates for cell culture were pre-coated with PLL solution in room temperature for at least 15 min, prior to cell seeding. Approximately 2.1 × 10^4^ cells were seeded on each substrate under standard cell culture conditions at 37 ℃ in 5% CO_2_. Media was changed every 2 days. Cell growth was assessed using the CCK-8 assay. For analysis of cell morphology, cells were cultured for 7 d and, then fixed with 10% neutral buffered formalin (NBF) for 15 min at 25 ℃, and permeabilized with 0.3 % Triton X-100 solution for 5 min at room temperature. The fixed cells were stained with phalloidin (Life Technologies, Delhi, India) and Hoechst 33342 (Sigma-Aldrich, MI, USA), and visualized using fluorescence microscopy to confirm cell morphological features and spreading of NSCs. The phalloidin-stained area in the fluorescence images was used to calculate cell spreading on each substrate using image J software. To assess cell adhesion, cells were cultured for 7 d and stained with vinculin (R&D Systems, MN, USA). To quantify the level of vinculin in cells, real-time RT-qPCR was conducted.

### 3.4. Neural Differentiation and Confirmation of mNSCs

After cells reached 70–80% confluency in culture media, 10^−6^ M RA was added to induce neural differentiation. Cells were maintained in differentiation media for 3 days. Media for cell cultivation were changed every 2 days. Neural differentiation of mNSCs was analyzed with immunofluorescence imaging and RT-qPCR. For immunofluorescence analysis of neurogenesis, cells were cultured for 14 days prior to the addition of primary antibodies nestin and GFAP, and subsequently incubated for 3 h at room temperature and overnight at 4 °C, respectively. Thereafter, NorthernLights-conjugated anti-mouse IgG emitting 493 nm and Alexa Fluor-conjugated anti-mouse IgG emitting 568 nm, which bind to primary antibodies nestin and GFAP, were added prior to an additional 1 h of incubation. NorthernLights-conjugated anti-mouse IgG emitting 557 nm for Class III β-tubulin were incubated for 3 h, and the nuclei of cells were stained with Hoechst. RT-qPCR was prepared in order of cell lysis, RNA extraction, and cDNA synthesis. Subsequently, qPCR was performed with SYBR Premix Ex Taq (Takara) in StepOnePlus. The threshold cycle values (ΔCt) from RT-qPCR results were calculated as fold changes on the basis of the Livak method (2^−ΔΔCt^); RT-qPCR results were normalized to the gene level of GAPDH as a house keeping gene.

### 3.5. Statistical Analysis

All quantitative data were presented as the mean ± standard deviation (SD) with three replicates. Statistically significant differences between two groups were calculated using unpaired Student′s *t*-test. One way analysis of variance (ANOVA) was used to calculate statistical significances among multiple groups. In all data analyzed in this study, a value of *p* < 0.05 was considered significant.

## 4. Conclusions

In this study, the effects of a homogeneous nanostructure on neuronal differentiation were confirmed using a HNPA platform fabricated by LIL processes. The HNPAs exhibited an increase in cell adhesion and spreading, and neuronal differentiation. Specifically, cell spreading of mNSCs was enhanced by approximately 25%, compared to non-patterned groups. The proliferation and cell adhesion were enhanced, compared to non-patterned groups. According to the amount and size of neurospheres, the HNPA-500 nm provided better performance for facilitating neurosphere formation. Moreover, through several differentiation assays, we concluded that HNPAs are excellent candidates for selective and enhanced neuronal differentiation of NSCs. In future work, this platform can be applied to improve differentiation of various sources of stem cells (e.g., embryonic stem cells, induced pluripotent stem cells, and other adult stem cells) and the development of stem cell-based therapy for neurodegenerative diseases.

## Supplementary Materials

Supplementary materials can be found at https://www.mdpi.com/1422-0067/21/1/191/s1.

## Abbreviations

mNSC	Mouse neural stem cell
RA	Retinoic acid
HNPAs	Homogeneous nanohole pattern arrays
ECM	Extracellular matrix
CNS	Central neural system
LIL	Laser interference lithography
ITO	Indium tin oxide
PR	Photoresist
Abs	Absorbance
TuJ1	Class III beta-tubulin
NeuroD1	Neurogenic differentiation 1
MAP2	Microtubule-associated protein 2
