# Supplementary material for: Enhancing Neurogenesis of Neural Stem Cells Using Homogeneous Nanohole Pattern-Modified Conductive Platform
Source: Int J Mol Sci. 2019 Dec 26;21(1):191. doi: 10.3390/ijms21010191 (PMC6981825; doi:10.3390/ijms21010191)
Supplement: Supplementary file 1 [file ijms-21-00191-s001.pdf]

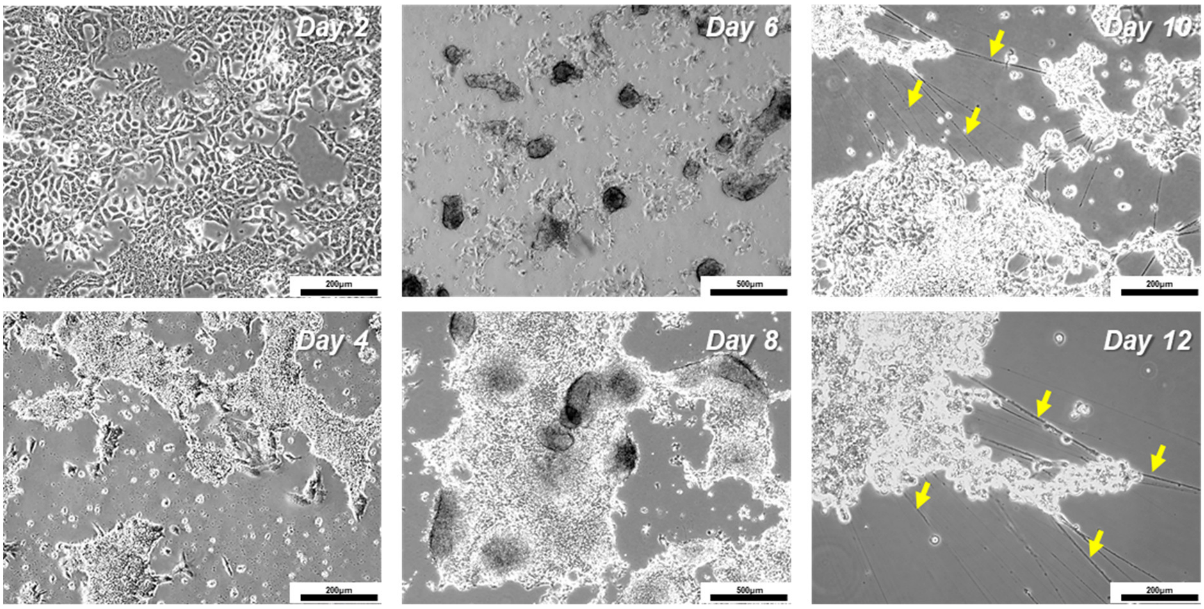

Supplementary Figure S1. Confirmation of neurite of mNSCs cultured on control group for 12 d with optical microscopic images. (yellow arrows: neurite)
